# Targeting the Interaction between the SH3 Domain of Grb2 and Gab2

**DOI:** 10.3390/cells9112435

**Published:** 2020-11-07

**Authors:** Francesca Malagrinò, Antonio Coluccia, Marianna Bufano, Giuseppe La Regina, Michela Puxeddu, Angelo Toto, Lorenzo Visconti, Alessio Paone, Maria Chiara Magnifico, Francesca Troilo, Francesca Cutruzzolà, Romano Silvestri, Stefano Gianni

**Affiliations:** 1 Istituto Pasteur—Fondazione Cenci Bolognetti, Dipartimento di Scienze Biochimiche “A. Rossi Fanelli” and Istituto di Biologia e Patologia Molecolari del CNR, Sapienza Università di Roma, 00185 Rome, Italy; francesca.malagrino@uniroma1.it (F.M.); angelo.toto@uniroma1.it (A.T.); lorenzo.visconti@uniroma1.it (L.V.); alessio.paone@uniroma1.it (A.P.); maria.magnifico@uniba.it (M.C.M.); francesca.troilo@uniroma1.it (F.T.); francesca.cutruzzola@uniroma1.it (F.C.); 2 Laboratory affiliated to Istituto Pasteur Italia—Fondazione Cenci Bolognetti, Dipartimento di Chimica e Tecnologie del Farmaco, Sapienza Università di Roma, Piazzale Aldo Moro 5, 00185 Rome, Italy; antonio.coluccia@uniroma1.it (A.C.); marianna.bufano@uniroma1.it (M.B.); giuseppe.laregina@uniroma1.it (G.L.R.), michela.puxeddu@uniroma1.it (M.P.); romano.silvestri@uniroma1.it (R.S.)

**Keywords:** Gab2, SH3 domain, virtual screening, kinetics, cancer cell lines

## Abstract

Gab2 is a scaffolding protein, overexpressed in many types of cancers, that plays a key role in the formation of signaling complexes involved in cellular proliferation, migration, and differentiation. The interaction between Gab2 and the C-terminal SH3 domain of the protein Grb2 is crucial for the activation of the proliferation-signaling pathway Ras/Erk, thus representing a potential pharmacological target. In this study, we identified, by virtual screening, seven potential inhibitor molecules that were experimentally tested through kinetic and equilibrium binding experiments. One compound showed a remarkable effect in lowering the affinity of the C-SH3 domain for Gab2. This inhibitory effect was subsequently validated in cellula by using lung cancer cell lines A549 and H1299. Our results are discussed under the light of previous works on the C-SH3:Gab2 interaction.

## 1. Introduction

The Grb2-associated binding protein (Gab2) is a scaffolding protein that organizes several signaling pathways, acting as a platform for the assembly of complex interaction networks [1]. In response of specific stimuli from the membrane, Gab2 becomes activated – an event that triggers the interaction with diverse targets controlling critical cellular processes, such as proliferation, migration, and differentiation [2,3,4]. Thus, while lacking any enzymatic functions, Gab2 works by transmitting and amplifying several signals downstream, in response to specific effectors. 

Gab2 was found overexpressed in breast [5], gastric [6] and lung [7] cancers. Furthermore, its role is clearly established in hematological cancers, such as juvenile myelomonocytic leukemia [8], chronic myelogenous leukemia (CML) [9], acute leukemia [10] and acute lymphoblastic leukemia [11]. Gab2 knock out mice models, although displaying a deficient allergic response and mast cells developments, are viable and fertile [9] and have proven to be a precious tool to pinpoint the role of this protein in cancer at a molecular level. It was observed GAB2 knock out mice are resistant to CML-like myeloproliferative neoplasms induced by BCR-ABL [9], a constitutively active fusion tyrosine kinases, capable of transforming hematopoietic stem cells, which is generated in CML by a translocation between chromosome 9 and 22; t(9;22)(q34;q11). BCR-ABL binds Gab2, which then activate distinct pathways involved in promoting leukemogenesis, including RAS-RAF-MEK-ERK, PI3K-AKT, and JAK-STAT [12]. Gab2 is therefore required for cell transformation by BCR-ABL.

Among its interaction partners, the protein growth factor receptor-bound protein 2 (Grb2) plays an important physiological role [2], which is at the basis of cell differentiation and proliferation. Grb2 represents a fundamental adapter protein in recruiting and assembling multimeric signaling complexes that are at the basis of crucial molecular pathways [13]. The interaction between Grb2 and Gab2 typically occurs in response to specific external stimuli and represents one of the first steps of the activation of the Ras/Erk pathway, tuning critical cellular processes such as proliferation, migration, and transformation [13,14]. An impaired interaction between Gab2 and Grb2 is implicated in the onset and progression of different types of cancers [1,4], thus being a valuable potential anticancer drug target.

From a molecular perspective, Gab2 is composed by a terminal Pleckstrin Homolgy-domain covalently attached to a long disordered region, constituted by several consensus motifs and binding sites recognized by specific protein-protein interaction domains [1,2,4,15]. Thus, while the major part of the protein is disordered, this portion is nevertheless functional and serves as a multiple binding site for several specific partners. Grb2 binds Gab2 by recognizing a specific RxxK consensus sequence of Gab2, within a proline-rich region encompassing residues 503 to 524 of Gab2, through its C-terminal SH3 domain (C-SH3) [16]. Because of its importance in cell proliferation and transformation, it is tempting to speculate that an effective chemotherapeutic strategy would be that of interfere with the binding between the C-SH3 domain of Grb2 and Gab2. By following these premises, here we report the successful identification and characterization of a lead compound, inhibiting this critical protein-protein interaction. The molecule was originally selected by virtual screening and subsequently produced by chemical synthesis. As described below, both in vitro and in cellula experiments confirm that the lead compound successfully interfere with the binding between the C-SH3 domain of Grb2 and a peptide mimicking the 503 to 524 sequence of Gab2 and highlight its capability to inhibit the growth of cancer cell lines.

## 2. Materials and Methods

### 2.1. Chemistry

Compounds AN-153-I158560 and AN-465-J137-985 were purchased from Life Chemicals Europe GmbH (Unterhaching, Germany) (Figure S1, Supporting Information). Compounds F0526-1467, F2096-1321, F5030-1061, F5139-0164 and F6599-2263 were purchased from Specs (Zoetermeer, The Netherlands) (Figure S1, Supporting Information). All reagents and solvents were handled according to the material safety data sheet of the supplier and were used as purchased without further purification. Organic solutions were dried over anhydrous sodium sulfate. Evaporation of solvents was carried out on a Büchi Rotavapor R-210 equipped with a Büchi V-850 vacuum controller and a Büchi V-700 vacuum pump. Column chromatography was performed on columns packed with silica gel from Merck (70−230 mesh). Silica gel thin layer chromatography (TLC) cards from Merck (silica gel precoated aluminum cards with fluorescent indicator visualizable at 254 nm) were used for TLC. Developed plates were visualized with a Spectroline ENF 260C/FE UV apparatus. Melting points (mp) were determined on a Stuart Scientific SMP1 apparatus and are uncorrected. Infrared (IR) spectra were recorded on a PerkinElmer Spectrum 100 FT-IR spectrophotometer equipped with a universal attenuated total reflectance accessory and IR data acquired and processed by PerkinElmer Spectrum 10.03.00.0069 software. Band position and absorption ranges are given in cm^−1^. Proton nuclear magnetic resonance (^1^H NMR) spectra were recorded with a Bruker Avance (400 MHz) spectrometer in the indicated solvent, and the corresponding fid files were processed by MestreLab Research SL MestreReNova 6.2.1-769 software. Chemical shifts are expressed in δ units (ppm) from tetramethylsilane. Compound purity was checked by high pressure liquid chromatography (HPLC). Purity of tested compounds was found to be >95%. The HPLC system used (Thermo Fisher Scientific Inc., Waltam, MA, USA, Dionex UltiMate 3000) consisted of an SR-3000 solvent rack, a LPG-3400SD quaternary analytical pump, a TCC-3000SD column compartment, a DAD-3000 diode array detector, and an analytical manual injection valve with a 20 μL loop. Samples were dissolved in acetonitrile (1 mg/mL). HPLC analysis was performed by using a Thermo Fisher Scientific Inc. Acclaim 120 C18 column (5 μm, 4.6 mm × 250 mm), at 25 ± 1 °C with an appropriate solvent gradient (acetonitrile/water), flow rate of 1.0 mL/min and signal detector at 206, 230, 254 and 365 nm. Chromatographic data were acquired and processed by Thermo Fisher Scientific Inc. Chromeleon 6.80 SR15 Build 4656 software.

### 2.2. Chemical Synthesis of N-(4-((3-Chlorobenzyl)oxy)-3-ethoxybenzyl)-2-(4-methoxyphenyl)ethan-1-amine (AN-465-J137-985)

To validate the inhibitory activity of compound AN-465-J137-985 we resynthesized the molecule and reproduce our experiments. The results matched unambiguously the ones obtained with the purchased AN-465-J137-985 compound. The chemical synthesis of compound AN-465-J137-985 was depicted in Scheme S2 (Supporting Information). Briefly, the reaction of 1-chloro-3-(chloromethyl)benzene with 3-ethoxy-4-hydroxybenzaldehyde in *N*,*N*-dimethylformamide in the presence of cesium carbonate at 70 °C for 1 h furnished 4-((3-chlorobenzyl)oxy)-3-ethoxybenzaldehyde. The latter was reacted with 2-(4-methoxyphenyl)ethan-1-amine in methanol in the presence of sodium borohydride at 25 °C for 12 h and then in the presence of 1N sodium hydroxide aqueous solution at 25 °C for 2 h to give *N*-(4-((3-chlorobenzyl)oxy)-3-ethoxybenzyl)-2-(4-methoxyphenyl)ethan-1-amine (AN-465-J137-985).

3-Ethoxy-4-hydroxybenzaldehyde (0.100 g; 0.602 mmol) was dissolved in anhydrous *N*,*N*-dimethylformamide (4 mL), then 3-chloro benzyl chloride (0.116 g, 0.722 mmol) and cesium carbonate (0.294 g, 0.902 mmol) were added. The reaction mixture was heated at 70 °C for 1 h under argon stream, cooled, diluted with water, and extracted with ethyl acetate. The organic layer was washed with brine, dried, and concentrated to give 4-((3-chlorobenzyl)oxy)-3-ethoxybenzaldehyde (0.170 g, 97%), pf 37–40 °C (from ethanol). ^1^H NMR (DMSO-*d_6_*): δ 1.36 (t, *J* = 7.0 Hz, 3H), 4.12 (q, *J* = 7.0 Hz, 2H), 5.26 (s, 2H), 7.24 (d, *J* = 8.3 Hz, 1H), 7.42–7.45 (m, 4H), 7.51–7.54 (m, 2H), 9.83 ppm (s, 1H). IR: ν 1691 cm^−1^. To a solution of 4-((3-chlorobenzyl)oxy)-3-ethoxybenzaldehyde (0.100 g, 0.344 mmol) and 2-(4-methoxyphenyl)ethan-1-amine (0.109 g, 0.723 mmol) in methanol (1 mL) at 0 °C was added sodium borohydride (0.025 g, 0.654 mmol). The reaction mixture was stirred at 0 °C for 30 min. The ice-water bath was removed and the reaction was stirred at 25 °C for 12 h; the mixture was concentrated and the residue was stirred in the presence of 1N sodium hydroxide aqueous solution (2 mL) for 2 h. Dichloromethane was added and layers were separated. The organic one was washed with brine, dried, and filtered. Evaporation of the solvent gave a residue that was purified by column chromatography (silica gel, ciclohexane:ethyl acetate = 7:3) to furnish *N*-(4-((3-chlorobenzyl)oxy)-3-ethoxybenzyl)-2-(4-methoxyphenyl)ethan-1-amine (AN-465-J137-985) as an oil (0.040 g, 30%). ^1^H NMR (DMSO-*d_6_*): δ 1.32–1.36 (m, 5H), 4.00–4.06 (m, 3H), 4.39–4.40 (m, 3H), 5.06 (t, *J* = 5.7 Hz, 2H), 5.10 (s, 3H), 6.77–6.80 (m, 2H), 6.93–6.96 (m, 3H), 7.35–7.43 (m, 5H), 7.50–7.52 ppm (m, 2H). IR: ν 3360 cm^-1^. While the stability of the molecule was found to be very high at room temperature, in the presence of DMSO, and the molecule retained its activity for weeks when stored at room temperature, we did not evaluate the serum stability of the molecule yet. In fact, as explained below, future work based on the chemical modification of the molecule will be needed to further improve the K_D_, before the molecule could be considered to be a valuable potential drug.

### 2.3. Molecular Modeling Studies

All molecular modeling studies were performed on a MacPro dual 2.66 GHz Xeon running Ubuntu 14 LTS. The Grb2/Gab2 structures were downloaded from the PDB, pdb code 2W0Z and 2VWF [16]. Hydrogen atoms were added to the protein, using Maestro protein preparation wizard [17]. Ligand structures were built with Maestro and minimized using the MMFF94x force field until a rmsd gradient of 0.05 kcal/(mol·Å) was reached. The docking simulations were performed using PLANTS. [18] We set a binding lattice of 12 Å radius using all default settings used. The pharmacophore model was obtained by Phase. [19] The polar features had a tolerance of 2 Å while the hydrophobic features had a tolerance of 2.5 Å. The commercially available compounds library (Maybridge, [www.Maybridge.com] Specs [www.SPECS.net] and Lifechiemical [www.lifechemicals.com] about 1,800,000 derivatives) was firstly filtered out by the rule of five, [20] then the obtained training set (about 1,400,000) was docked at the Grb2 C-SH3 domain. All the docking proposed binding conformation (10 per molecule) were filtered out by the pharmacophore model and the 50 first ranked derivatives were visual inspected. The images in the manuscript were created with PyMOL [21].

### 2.4. Expression and Purification of C-SH3 Grb2

C-SH3 Grb2 was expressed and purified as previously reported [22].

### 2.5. Kinetic Binding Experiments

Kinetic experiments were performed on a single-mixing SX-18 stopped-flow instrument (Applied Photophysics). The excitation wavelength used was 280 nm while the fluorescence emission was collected using a 320-nm cut-off glass filter. The binding experiments were carried out at 20 °C in pseudo-first order condition mixing a constant concentration of C-SH3 Grb2 (2 µM)—in absence (control) and in presence of a constant concentration of AN-465-J137-985 (5 µM)—versus increasing concentrations of Gab2_503–524_ (ranging from 1 to 12 µM). Displacement experiments were carried out challenging a preincubated complex composed by C-SH3 and dansyl-Gab2_503–524_ (2 µM, molar ratio 1:1)—in absence (control) and in presence of 5 µM AN-465-J137-985—versus a high excess of Gab2 (80 µM). All observed rate constants (*k_obs_*) were calculated from the average of 3–6 single traces. All binding and displacement time courses were satisfactorily fitted by a single exponential equation. For all measurements the buffer used was HEPES 50 mM, 0.5 M NaCl, pH 7.0, 20% *v/v* DMSO.

### 2.6. Equilibrium Binding Experiments

Fluorescence equilibrium binding experiments were performed on the FluoroMax-4 single photon counting spectrofluorometer (Horiba). The excitation wavelength used was 280 nm while the fluorescence emission was recorded between 300 and 400 nm. The experiments were performed at 25 °C using a quartz cuvette with a path length of 1 cm. For all measurements the buffer used was HEPES 50 mM, 0.5 M NaCl, pH 7.0, 20% *v/v* DMSO. The experiments were carried out by mixing a constant concentration of C-SH3 (2 µM)—in absence (control) and in presence of a constant concentration (5 µM) of the molecule to be tested—versus increasing concentrations of Gab2 (ranging from 1 to 38 µM). The potential inhibitory molecules tested were AN-153-I158560, F0526-1467, F2096-1321, F5030-1061, F5139-0164, F6599-2263 AN-465-J137-985 (Figure S1, Supporting Information). The dependences of the normalized fluorescence signals at 330 nm at different concentration of Gab2 were satisfactorily fitted with the following hyperbolic equation:

(1) $$F_{y}=A\frac{\left[Gab2^{*}\right]}{\left[Gab2^{*}\right]+K_{D}}$$

where *F*_y_ represents the observed fluorescence and *A* is the transition amplitude.

### 2.7. Cell Lines

A549 and H1299 cancer cells were grown in RPMI-1640 medium supplemented with 2 mM L-glutamine, 100 IU mL^−1^ penicillin/streptomycin and 10% fetal calf serum (Sigma-Aldrich). In analogy to the in vitro experiments, in all experiments in cellula, AN-465-J137-985 has been solubilized in DMSO and, analogously, all the control experiments have been performed in identical conditions.

### 2.8. Trypan Blue Exclusion Assay

A549 and H1299 cell lines were treated with increasing doses of AN-465-J137-985 for 48 h. After the incubation, the cells have been harvested and, following the addition of a 0.4% (*w/v*) trypan blue solution (Sigma-Aldrich), were transferred to a Burker counting chamber (Hirschmann, Germany) and visualized using an Axioskop 2 plus micro-scope (Carl Zeiss Microscopy, Switzerland). Cells stained with trypan blue dye were considered nonviable.

### 2.9. Propidium Iodide (PI) Staining

The growth medium of A549 and H1299 cells, incubated with AN-465-J137-985 and/or transfected with the plasmid encoding for the C-SH3 fragment of Grb2 or a control plasmid (PCDNA) for 48 h, was collected and the cells were washed once with phosphate buffered saline (also collected). Adherent cells were harvested, and all the collected cell fractions were centrifuged 5 min at 1000 rpm. Detached cells were washed with cold PBS twice, and fixed in 70% ethanol for 30 min. After another wash with PBS, cells were incubated with 1 µg mL^−1^ PI for 20 min at 25 °C before cytometric analysis by BD Accuri C6 Flow Cytometer (Becton Dickinson, Franklin Lakes, NJ, USA). Cells were considered apoptotic when their DNA content was <2n (sub-G1 cell population).

### 2.10. Annexin-V-FITC Staining

Detection of apoptotic cells was performed by flow cytometry using Apoptosis Assay Kit (Cat. No. IK-11120). Briefly, A549 and H1299 cells, treated with AN-465-J137-985 and/or transfected with the plasmid encoding for the C-SH3 fragment of Grb2 or a control plasmid (PCDNA) for 24 h and/or 48 h, were harvested, washed once with cold PBS, resuspended in Binding Buffer at a concentration of 1 × 10^6^ cells mL^−1^ and incubated with Annexin-V-FITC and propidium iodide for 15 min at room temperature. The cytometric analysis was performed by BD Accuri C6 Flow Cytometer (Becton Dickinson, Franklin Lakes, NJ, USA), using 488 nm excitation and a 515 nm band pass filter for FITC detection and a filter > 600 nm for PI detection. To set up compensation and quadrants, the following controls are used: unstained cells; cells stained with FITC Annexin-V (no PI) and cells stained with PI (no FITC Annexin-V).

### 2.11. RNA Extraction and Real-Time qRT-PCR Analyses

Total RNA was extracted using TRIzol reagent (Invitro-gen) following the manufacturer’s instructions. About 1 µg of RNA was used for the reverse transcription reaction by using a SuperScript First-Strand Synthesis System Kit (Invitrogen). The cDNA was used for qRT-PCR analysis (in triplicate for each sample) using KAPA Sybr Fast universal qPCR Kit following the manufacturer’s instructions. Reactions were performed by Stratagene MX3000P (Stratagene, La Jolla, CA, USA).

### 2.12. Statistical Analysis

Statistical analysis was performed using one way ANOVA followed by the Bonferroni post-hoc comparison test. *p* < 0.05 was considered significant.

## 3. Results and Discussion

### 3.1. Identifying Binding Inhibitors by Virtual SCREENING

To identify valuable compounds able to impair the interaction between C-SH3 and Gab2 interaction we screened by docking and pharmacophore model commercially available databases (Maybridge, Specs and LifeChemicals). The whole training set (about 2,000,000 derivatives) was first filtered by the Lipinski Rule of Five [20] compliance to ensure druglike property of the selected compounds. The obtained training set was docked by Plants to Grb2 C-SH3 domain.

**Figure 1:**
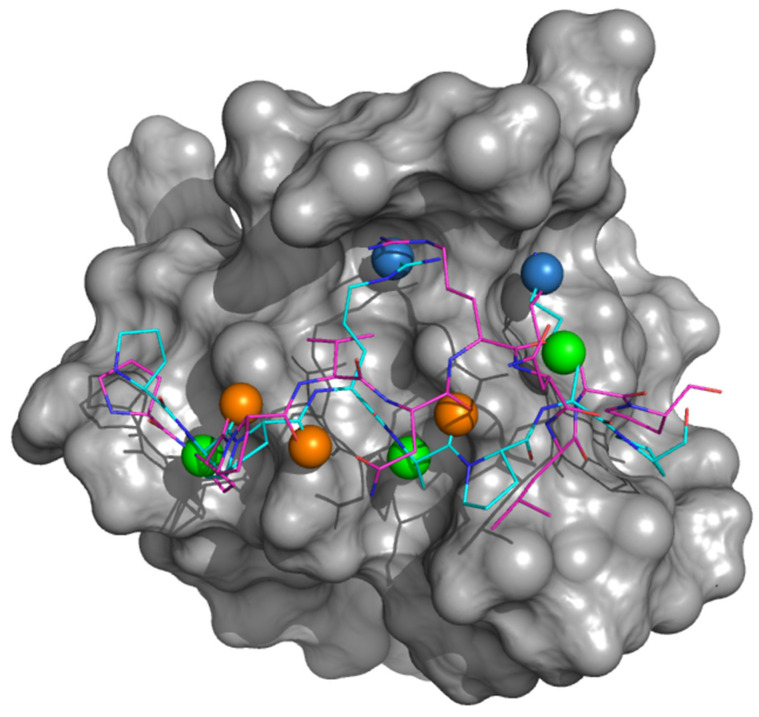
Pharmacophore model for Grb2 C-SH3:Gab2 complex. The Grb2 C-SH3 domain is reported as gray surface. The Gab2 core binding regions are reported as lines, Gab2a PPPRPPKP cyan and Gab2b PPVNRNLKP magenta. The pharmacophore queries are depicted as sphere: green for hydrophobic, orange for H-bond acceptor and light blue for positive charge and H-bond donor.

The Gab2 core binding regions (Gab2a PRxxK pdb code 2W0Z and Gab2b VNRxxK pdb code 2VWF) [16] were used to draw a pharmacophore model that gathered the pharmacophoric interactions of Gab2 to its target. (Figure 1). All the docking proposed poses were ranked by the fitting to the pharmacophore model. The best compounds were visually inspected and seven derivatives (AN-153-I158560, F0526-1467, F2096-1321, F5030-1061, F5139-0164, F6599-2263 and AN-465-J137-985) were selected for the further analyses.

**Figure 2:**
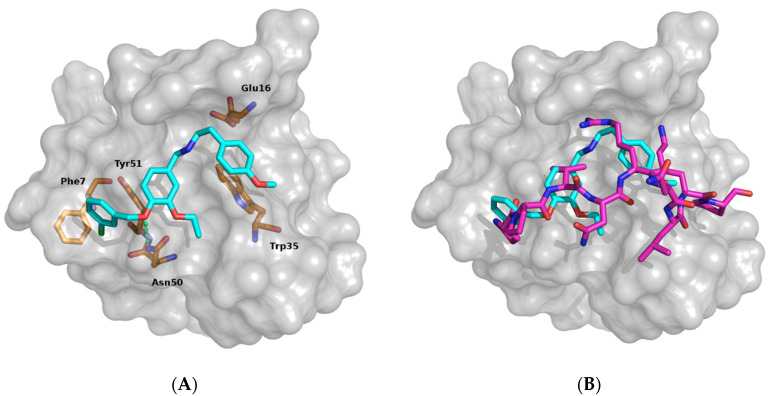
AN-465-J137-985 proposed binding mode. (**A**) Proposed binding mode for derivative AN-465-J137-985. The Grb2 C-SH3 domain is reported as gray surface. AN-465-J137-985 is reported as cyan stick; residues involved in contacts are reported as light green. H-bond is depicted as yellow dot lines. (**B**) Superimposition of AN-465-J137-985 proposed binding and Gab2a core binding. Importantly, in the absence of specific experimental data, the proposed binding mode should be considered solely as a plausible hypothesis.

As detailed below, out of these seven compounds, experiments confirmed an inhibiting binding capability only in the case of AN-465-J137-985. Hence, in an effort to provide additional insights and to elucidate its properties, we resorted to characterize computationally the structural featured of its binding to C-SH3. In particular, the analyses of the docking proposed AN-465-J137-985 binding mode led us to identify a series of crucial contacts with its cognate target, reported in Figure 2. We observed T-shaped aromatic interactions between the chlorobenzyl ring with Phe7 and between the central phenyl ring with Tyr51 (Figure 2A). Another aromatic interaction involved the methoxyphenyl moiety and the Trp35. We highlighted also two polar contacts, one involving the secondary amine function and the carboxylic moiety of the Glu16, the second polar contact was a H-bond between the C-SH3 Asn50 amide moiety with the ether oxygen atom bridging the two aromatic rings (Figure 2A). The superimposition of the AN-465-J137-985 proposed binding and Gab2a core binding showed as: the secondary amine moiety laid in the same zone of the Arg515 side chain being able to do similar contacts, the three aromatic rings were superimposable with Pro512 and Val^513^ side chains and Arg515 backbone mimicking the hydrophobic contact observed for the substrate. Lastly the ether oxygen atom between the aromatic rings is superimposable with the Pro^512^ amide oxygen atom doing the same polar contact. (Figure 2B).

### 3.2. Testing the Inhibitor Compounds In Vitro

**Figure 3:**
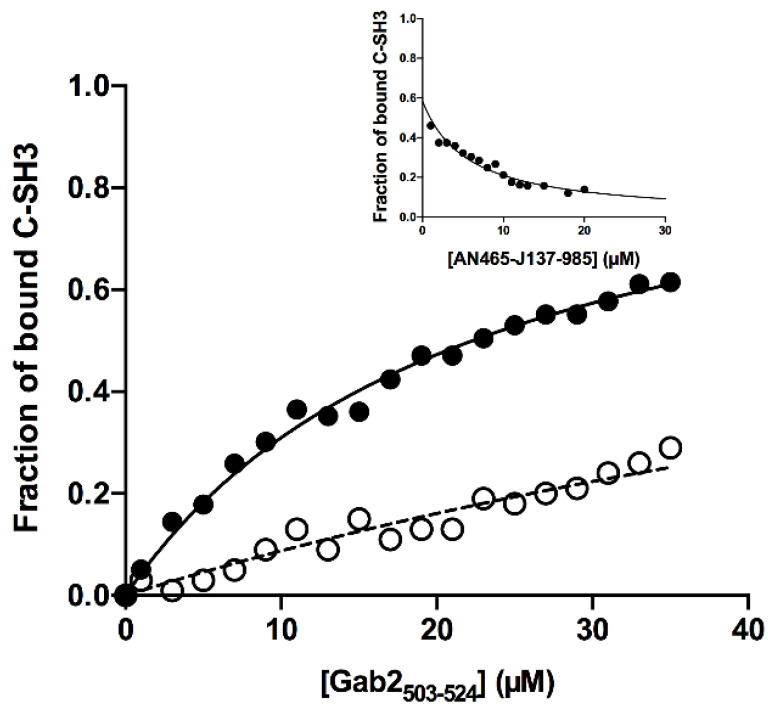
Equilibrium binding titration monitored by change in intrinsic fluorescence emission of C-SH3 at different concentrations of Gab2* in absence (full circles) and in presence of 5 µM AN-465-J137-985 (empty circles). Continuous and broken lines are the best fit to a hyperbolic function. Inset panel. Titration of the complex between C-SH3 and Gab2*, a constant concentration of 1 µM and 25 µM respectively, with varying concentrations of AN-465-J137-985.

To confirm experimentally whether the seven derivatives AN-153-I158560, F0526-1467, F2096-1321, F5030-1061, F5139-0164, F6599-2263 and AN-465-J137-985 could efficiently target the binding between the C-SH3 domain of Grb2 and Gab2 we resorted to perform equilibrium binding experiments. In analogy to our previous works [22,23], we designed the experiments by challenging the C-SH3 domain with a peptide mimicking a specific region of Gab2, ranging from residue 503 to 524 (Gab2*). By taking advantage of the two naturally occurring tryptophan residues of the C-SH3 domain in positions 35 and 36, the binding reaction was monitored by following the change of the intrinsic tryptophan fluorescence emission at increasing concentration of ligand. Experiments were conducted in buffer HEPES 50 mM, 0.5 M NaCl, pH 7.0 at 25 °C in the presence of 20% *v/v* DMSO, to ensure the solubility of the potential inhibitory molecules. To calculate the affinity between C-SH3 Grb2 and Gab2* in these experimental conditions we conducted a control experiment by challenging a constant concentration of C-SH3 (2 µM) versus increasing concentrations of Gab2* (ranging from 1 to 38 µM). The dependence of the normalized fluorescence signal at 330 nm at different concentration of Gab2* is reported in Figure 3.

Data were satisfactorily fitted with a hyperbolic equation, the calculated *K_D_* being 22 ± 2 µM (X^2^ = 0.9916). Thus, we resorted to test the inhibitory effect of the designed molecules by performing equilibrium binding experiments in the presence of the different molecules in solution at constant concentration of 5 µM. While the experiments conducted in the presence of molecules AN-153-I158560, F0526-1467, F2096-1321, F5030-1061, F5139-0164, F6599-2263 did not return any detectable change in the affinity of the C-SH3 domain for Gab2* (Figure S3), the equilibrium binding titration performed in the presence of AN-465-J137-985 displayed a clear effect on the binding reaction. The dependence of the normalized fluorescence signal at 330 nm at different concentration of Gab2* compared to the control experiment is reported in Figure 3. The calculated *K_D_* in the presence of AN-465-J137-985 at the concentration of 5 µM was 100 ± 15 µM (X^2^ = 0.9916), which corresponds to a decrease of affinity of C-SH3 for Gab2 of ~5 fold as compared to the data in the absence of inhibitor. This result clearly shows an inhibitory effect of the AN-465-J137-985 molecule on the binding between C-SH3 and Gab2. It should be noticed that while it is clear that the presence of AN-465-J137-985 induces a decrease in affinity between the C-SH3 domain and Gab2*, due to the low affinity between the interacting partners, it is extremely difficult to infer the inhibition quantitatively from equilibrium experiments only. To estimate the inhibition constant of AN-465-J137-985, we resorted to titrate a constant concentration of a pre-incubated complex involving C-SH3 and Gab2* with varying concentrations of AN-465-J137-985 (Figure 3, inset panel). As expected, the observed fluorescence decreases with increasing concentrations of AN-465-J137-985, indicating a decrease in affinity with an apparent inhibition constant of about 5 ± 1 µM.

**Figure 4:**
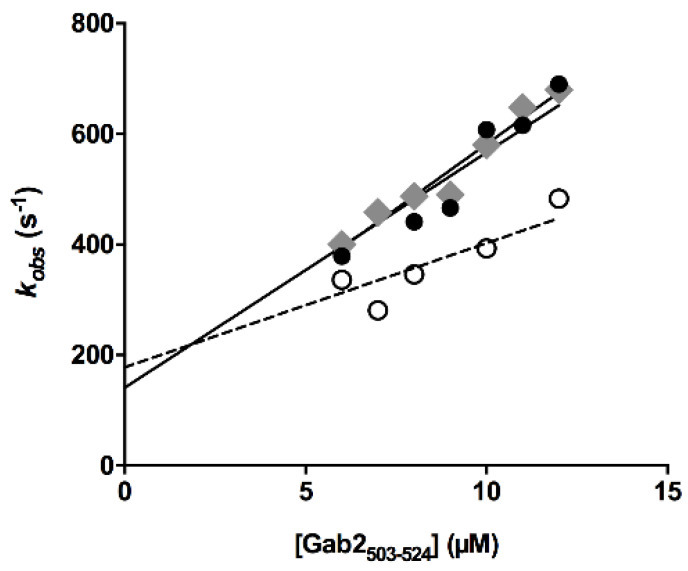
Observed rate constants measured through stopped-flow kinetic binding experiments by rapidly mixing a constant concentration of C-SH3 versus different concentrations of Gab2* in absence (full circles) and in presence of 5 µM AN-465-J137-985 (empty circles). The data recorded in the absence of DMSO and AN-465-J137-985 are reported in gray diamonds. Lines are the best fit to a linear equation.

To infer the mechanism whereby AN-465-J137-985 affects the C-SH3:Gab2 binding reaction, we resorted to performing pseudo-first order kinetic binding experiments. By taking advantage of a stopped-flow apparatus, a solution containing a constant concentration of the C-SH3 domain at the concentration of 2 µM was rapidly mixed with an excess of Gab2* at different concentrations ranging from 0 to 12 µM. All the traces recorded at different concentrations of Gab2* were satisfactorily fitted with a single exponential equation and the calculated observed rate constants *k_obs_* were plotted as a function of the concentration of Gab2* (Figure 4). The analysis of the linear dependence of the *k_on_* returned the value of the microscopic association rate constant *k_on_* = 40.3 ± 0.5 µM^−1^ s X^−1^, corresponding to the slope of the fitting line.

The analysis of the data reported in Figure 4 allows in theory to extrapolate the microscopic dissociation rate constant by estimating the intercept on the y-axis. However this procedure is generally prone to large errors and may lead to a miscalculation of *k_off_*. Thus, we resorted to measure directly *k_off_* by displacement experiments [24]. In particular, we challenged a preincubated complex of C-SH3 and a dansylated variant of Gab2* at molar ratio 1:1 and at fixed concentration of 2 µM versus a high excess of Gab2* (80 µM). In agreement with theory, the fluorescence change upon displacement displayed a single exponential behavior and was found insensitive to displacer concentration, with a calculated *k_off_* of 170 ± 10 s^−1^. Observed displacement time courses are reported in Figure S4.

To test the effect of the presence of the AN-465-J137-985 on binding kinetics we repeated stopped-flow binding and displacement experiments by adding the AN-465-J137-985 molecule at the fixed concentration of 5 µM to the solution containing C-SH3, for binding experiments, and to the solution containing the preincubated C-SH3: Gab2* complex for displacement experiments.

The calculated observed rate constants were plotted as function of the concentrations of Gab2* (Figure 2) and fitted with a linear equation, the *k_on_* being 20.6 ± 1.5 µM^−1^ s^−1^. Microscopic dissociation rate constant *k_off_* was directly measure by displacement experiment, being 190 ± 20 s^−1^. Importantly, these values are consistent with what can be extrapolated from the pseudo-first order data reported in Figure 4. It is of interest to notice that while the *k_off_* values in absence and in presence of AN-465-J137-985 are comparable, a decrease of *k_on_* by a factor of 2 is clearly appreciable. Taken together our results clearly demonstrate that while the dissociation rate constant of Gab2 by the C-SH3 domain is mostly unaffected by the presence of the AN-465-J137-985 molecule, there is a clear effect on the recognition event between the two molecules that leads to a decrease in the binding affinity.

### 3.3. Validating the Effect of AN-465-J137-985 in Cellula

To further investigate the effect of AN-465-J137-985 we used two lung cancer cell lines evaluating the potential anti-cancer effects of this molecule.

**Figure 5:**
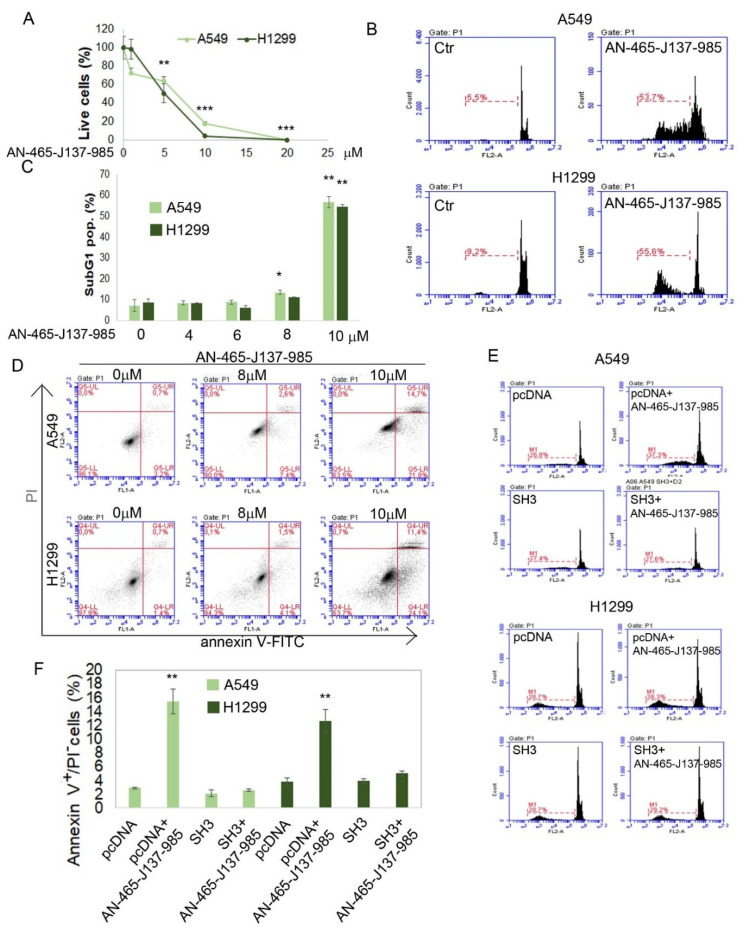
Effect of AN-465-J137-985 in A549 and H1299 lung cancer cells. (**A**) Evaluation of cell numbers after 48 h treatment with increasing concentrations of AN-465-J137-985 by trypan blue exclusion assay. B-C) PI staining of cells after 48 h incubation with increasing concentrations of AN-465-J137-985. (**B**) Representative experiments of A549 and H1299 treated or not with 10 µM AN-465-J137-985. The percentage of sub-G1 population is reported in red. (**C**) Average +/− standard deviation of the percentage of the Sub-G1 population. (**D**) Annexin-V/PI staining of cells 24 h after treatment with 8 and 10 µM AN-465-J137-985. (**E**) PI staining of cells 72h after transfection with the plasmid encoding for C-SH3 of GRb2 or a control plasmid (PCDNA) incubated for 48 h in presence or absence of AN-465-J137-985 8 µM. (**F**) Annexin-V/PI staining percentage of cells 48h after transfection with the plasmid encoding for C-SH3 of Grb2 or a control plasmid (PCDNA) incubated for 24 h in the presence or absence of AN-465-J137-985 8 µM. * *p* < 0.05, ** *p* < 0.01, *** *p* < 0.001. Each experiment have been repeated in triplicate.

We treated A549 and H1299 lung cancer cell lines with increasing doses of AN-465-J137-985 and evaluated cell survival using the trypan blue exclusion assay. The data in Figure 5A indicate that AN-465-J137-985 significantly inhibits the growth of both cancer cell lines indicating a lethal dose 50 (LD50) value of about 5 and 7 µM for H1299 and A549, respectively.

To better understand the mechanism that decreases cell survival we used propidium iodide (PI) and annexin-V/PI staining, in order to evaluate the possible activation of the apoptotic machinery. The cell cycle analysis reported in Figure 5B,C shows that AN-465-J137-985 at 10 µM concentration induces a strong accumulation of the cells in the sub-G1 population (around 50% for both cell lines) at 48 h after the treatment with respect to DMSO treated cells (Ctr), indicating the induction of a strong apoptotic effect.

Figure 5D shows annexin-V/PI staining of A549 and H1299 cells after the treatment with DMSO as control or with AN-465-J137-985 at 8 and 10 µM for 24 h. The drug-induced accumulation of the cells in the lower right quadrant (annexin-V positive/PI negative) confirm the hypothesis of the activation of the apoptotic mechanism.

To validate the selectivity of AN-465-J137-985, we overexpressed the C-SH3 fragment of Grb2 in both cell lines; the overexpression in the cells transfected with the plasmid was confirmed by RT-PCR (data not shown). The effect of 8 µM AN-465-J137-985 on cells transfected with the plasmid encoding for C-SH3 or a control plasmid (PCDNA) was evaluated 48 h after the transfection using PI (Figure 5E) and annexin-V/PI staining (Figure 5F) after 24 and 48 h treatment, respectively. Data in Figure 5E,F show that SH3 overexpression is able to rescue almost completely (~95%) the death effect induced in both cell lines by the treatment with AN-465-J137-985, indicating that C-SH3 fragment of Grb2 is a direct target of AN-465-J137-985. 

## 4. Conclusions

The interaction between the C-SH3 domain of Grb2 and Gab2 is of crucial importance for cell physiology and its implications for cancer onset and development are well known [4,5,6]. On these bases, it is of fundamental importance to develop a pharmaceutical anticancer strategy targeted to the inhibition of the binding of C-SH3 and Gab2. Structure-based drug design and screening is a powerful methodology, which can be employed to identify potential molecules able to interact with macromolecular targets. Interestingly, this strategy, combined with in vitro experiments, has been previously applied to the C-SH3:Gab2 protein system [25]. By employing a synergy between computational work and in vitro and in cellula experiments, we report here the successful identification of a molecule with a remarkable effect in lowering the affinity of the C-SH3 for Gab2. We also defined a protocol of chemical synthesis that paves the way for future modifications and improvement of the lead compound. It is of importance to stress how the inhibitory effect detected by equilibrium and kinetic binding experiment was successfully validated by two different types of cancer cell lines A549 and H1299, which confirms the anti-tumorigenic power of the molecule. Furthermore, while at this stage it is not possible to exclude cross-reactivity with other SH3 domains, the in cellula experiments performed with SH3 transfected cell lines appear to confirm the selectivity of AN-465-J137-985. Future experiments based on animal models as well as improvement of the observed K_D_ via chemical modification will further clarify this hypothesis.

## Supplementary Materials

The following are available online at https://www.mdpi.com/2073-4409/9/11/2435/s1, Figure S1: Chemical structures of compounds AN-153-I158560, F0526-1467, F2096-1321, F5030-1061, F5139-0164, F6599-2263 and AN-465-J137-985, Scheme S2: Chemical synthesis of AN-465-J137-985, Figure S3: Equilibrium binding in the presence of different inhibitors, Figure S4: Observed displacement time courses.
