# Supplementary material for: Targeting the Interaction between the SH3 Domain of Grb2 and Gab2
Source: Cells. 2020 Nov 7;9(11):2435. doi: 10.3390/cells9112435 (PMC7695167; doi:10.3390/cells9112435)
Supplement: Supplementary file 1 [file cells-09-02435-s001.pdf]

Supporting information for

Targeting the interaction between the SH3 domain of Grb2 and Gab2

Francesca Malagrinò 1, Antonio Coluccia 2, Marianna Bufano, 2 Giuseppe La Regina 2, Michela Puxeddu 2, Angelo Toto 1, Lorenzo Visconti 1, Alessio Paone 1, Maria Chiara Magnifico1,3, Francesca Troilo 1, Francesca Cutruzzolà 1, Romano Silvestri 2 and Stefano Gianni 1,*

[1] Istituto Pasteur - Fondazione Cenci Bolognetti, Dipartimento di Scienze Biochimiche "A. Rossi Fanelli" and Istituto di Biologia e Patologia Molecolari del CNR, Sapienza Università di Roma, 00185, Rome, Italy; francesca.malagrino@uniroma1.it (F.M.), angelo.toto@uniroma1.it (A.T.), lorenzo.visconti@uniroma1.it (L.V.), alessio.paone@uniroma1.it (A.P.), francesca.troilo@uniroma1.it (F.T.), francesca.cutruzzola@uniroma1.it (F.C.),

[2] Laboratory affiliated to Istituto Pasteur Italia – Fondazione Cenci Bolognetti, Dipartimento di Chimica e Tecnologie del Farmaco, Sapienza Università di Roma, Piazzale Aldo Moro 5, 00185 Rome, Italy; antonio.coluccia@uniroma1.it (A.C.), marianna.bufano@uniroma1.it (M.B), giuseppe.laregina@uniroma1.it (G.L.R.), michela.puxeddu@uniroma1.it (M.P.), romano.silvestri@uniroma1.it (R.S.).

[3] present address: Department of Biosciences, Biotechnologies and Biopharmaceutics, University of Bari "Aldo Moro", Via Orabona 4, 70121 Bari, Italy; maria.magnifico@uniba.it.

* Correspondence: stefano.gianni@uniroma1.it (S.G.)

Tables of contents

1. Figure S1. Chemical structures of compounds AN-153-I158560, F0526-1467, F2096-1321, F5030-1061, F5139-0164, F6599-2263 and AN-465-J137-985.

2. Scheme S2. Chemical synthesis of AN-465-J137-985.

3. Figure S3. Equilibrium binding in the presence of different inhibitors.

4. Figure S4. Observed displacement time-courses.

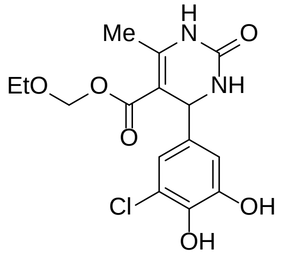

AN-153-I158560

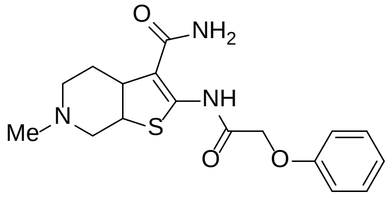

F0526-1467

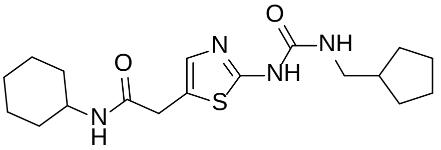

F2096-1321

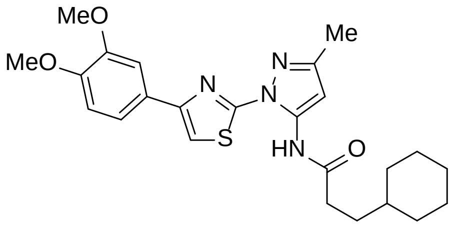

F5030-1061

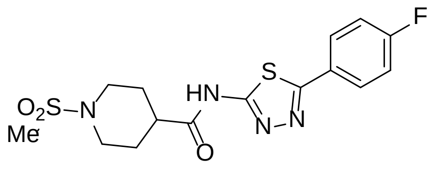

F5139-0164

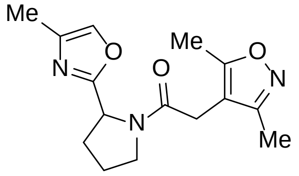

F6599-2263

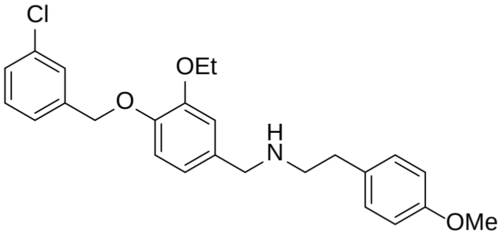

AN-465-J137-985

Figure S1. Chemical structures of compounds AN-153-I158560, F0526-1467, F2096-1321, F5030-1061, F5139-0164, F6599-2263 and AN-465-J137-985.

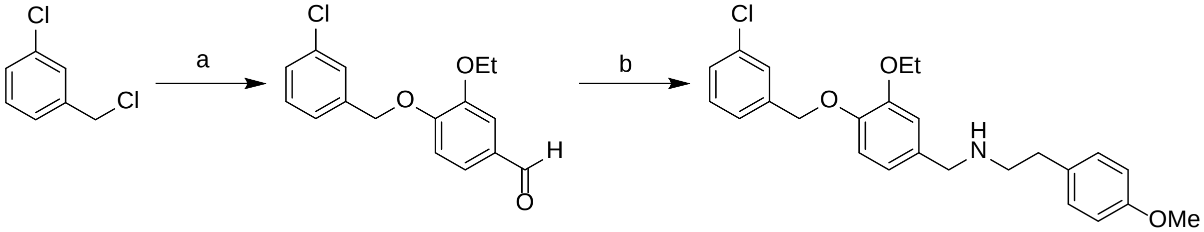

AN-465-J137-985

Scheme S2. Chemical Synthesis of AN-465-J137-985. Reagents and reaction conditions: (a) 3-ethoxy-4-hydroxybenzaldehyde, cesium carbonate, *N*,*N*-dimethylformamide, 70 °C, 1 h, argon stream, 97%; (b) (*i*) 2-(4-methoxyphenyl)ethan-1-amine, methanol, 0 °C, 30 min, then 25 °C, 12 h; (*ii*) 1N sodium hydroxide aqueous solution, 25 °C, 2 h, 30%.

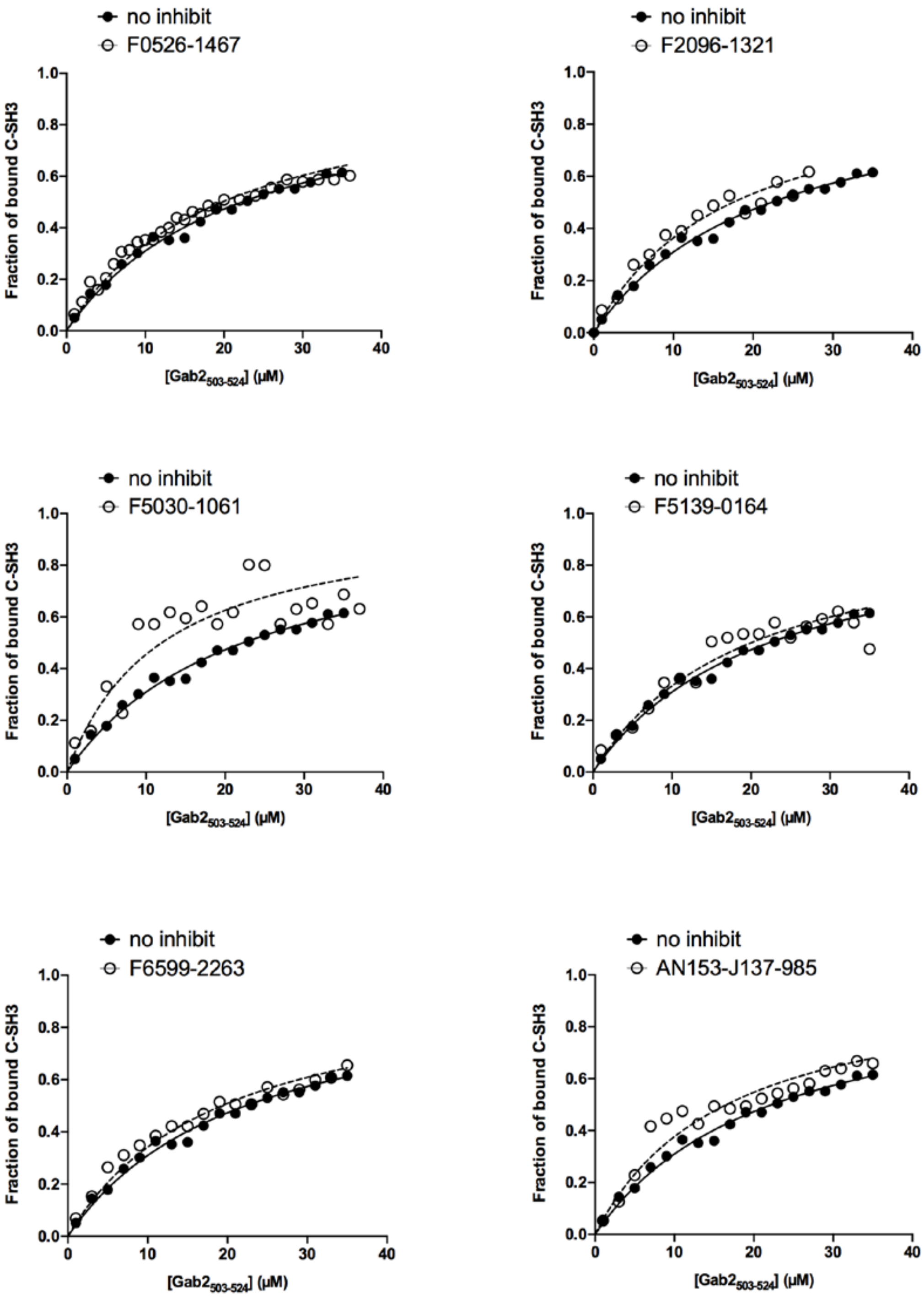

Figure S3. Equilibrium binding titration monitored by change in intrinsic fluorescence emission of C-SH3 at different concentrations of Gab2* in absence (full circles) and in presence of different inhibitors at a constant concentration of 5 μM.

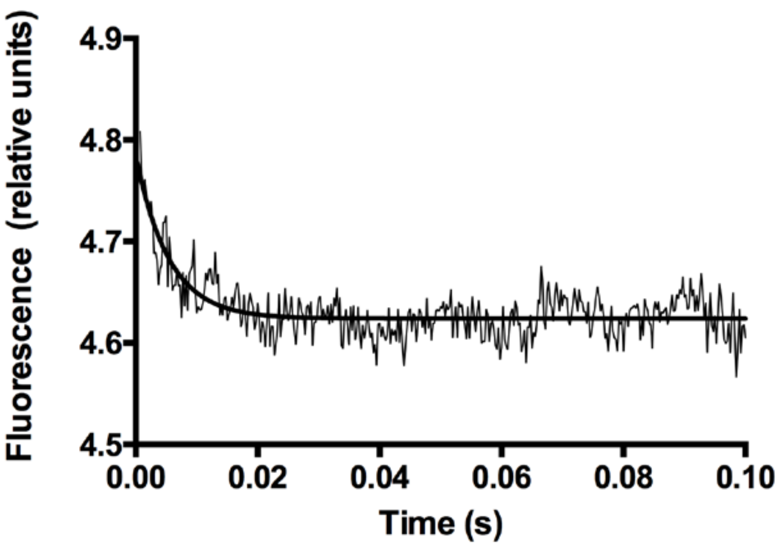

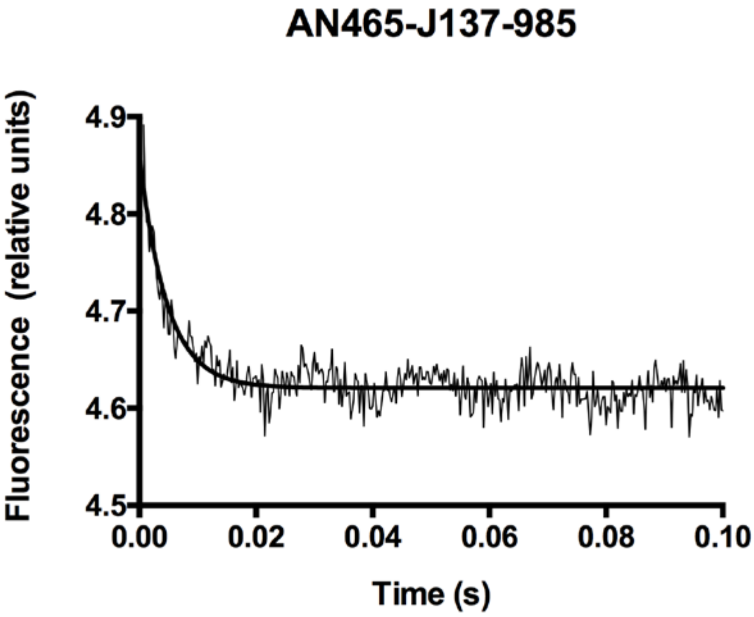

Figure S4. Observed displacement time-courses in the absence (top) and in the presence of 5μM AN-465-J137-985 (bottom). The experimental methodology and settings are described in the main text.
